# Esthetic, Functional, and Everyday Life Assessment of Individuals with Cleft Lip and/or Palate

**DOI:** 10.1155/2015/510395

**Published:** 2015-04-05

**Authors:** Nikolaos Gkantidis, Despina A. Papamanou, Marina Karamolegkou, Domna Dorotheou

**Affiliations:** ^1^Department of Orthodontics and Dentofacial Orthopedics, University of Bern, Freiburgstrasse 7, 3010 Bern, Switzerland; ^2^Department of Orthodontics, School of Dentistry, University of Athens, 2 Thivon Street, Goudi, 115 27 Athens, Greece; ^3^Department of Orthodontics, University of Geneva, Rue Barthélemy-Menn 19, 1205 Geneva, Switzerland

## Abstract

*Objectives*. To evaluate the level of satisfaction of individuals with cleft lip and/or palate (CLP) and their parents concerning the esthetic and functional treatment outcomes, the impact of the cleft on everyday life, and potential associations with treatment outcome satisfaction. *Subjects and Methods*. The sample consisted of 33 patients (7 CP, 20 unilateral CLP, and 6 bilateral CLP; median age: 17.1, range: 9.0–33.1 years) and 30 parents, who responded to a questionnaire in an interview-guided session. All participants received their orthodontic treatment at the Department of Orthodontics in the University of Athens. *Results*. Patients and their parents were quite satisfied with esthetics and function. Patients with UCLP primarily were concerned about nose esthetics (BCLP about lip esthetics and CP about speech). Increased satisfaction was associated with decreased influence of the cleft in everyday life (0.35 < rho < 0.64, *P* < 0.05). Parents reported significant influence of the cleft on family life, while patients did not. *Conclusions*. Despite the limited sample size of subgroups, the main concerns of patients with different cleft types and the importance of satisfying lip, nose, and speech outcomes for an undisturbed everyday life were quite evident. Thus, the need for targeted treatment strategies is highlighted for individuals with cleft lip and/or palate.

## 1. Introduction

Orofacial cleft, one of the most common congenital craniofacial anomalies, has a prevalence of approximately 1 in 700 live births [1]. Nonsyndromic clefts are generally divided into two categories: cleft palate (CP) and cleft lip and palate (CLP) with a prevalence of 0.031% and 0.091%, respectively [1]. The treatment of an individual with cleft lip and/or palate is a very difficult task and most of the time requires close long-term collaboration among various specialists. The multidisciplinary teamwork may eventually lead to a successful treatment outcome with a minimum of procedures and optimal cost-effectiveness [2]. However, it is quite usual that lack of long-term treatment planning from birth to adulthood and standardized surgical protocols result in poor esthetic and functional treatment outcomes [3–5].

The severity of cleft's nature and the long treatment duration may also have a great impact on the psychological and social development of patients and their parents [6]. Even though there is extensive research regarding the oral health related quality of life (OHRQL) of various patient types [7–9], patients with cleft appear not to be a favorable study group. Data collected from a mailed survey showed that there is a relationship between the ohrql and treatment satisfaction in individuals with cleft lip/palate and their parents [10]. Ward et al. [11] concluded that the presence of an orofacial cleft significantly decreases overall ohrql, functional well-being, and social-emotional well-being in children and adolescents, with similar impact in patients and parents.

Due to these considerations, the evaluation of patient's satisfaction from treatment, including possible associations between satisfaction from the esthetic and functional outcome with everyday life parameters, can offer valuable information to care providers. The investigation of the impact of a cleft on the social, professional, and family life of a patient relative to the esthetic and functional components of this specific condition may contribute to more successful and more targeted treatment approaches in the future. In the present study, we assessed the satisfaction of individuals with cleft lip and/or palate and their parents from the esthetic and functional treatment outcome. We used a diverse sample regarding cleft type and age/stage of treatment to explore the impact of the cleft on everyday life as well as potential associations of the esthetic and functional treatment outcome with everyday life parameters and other patient/treatment related characteristics.

## 2. Subjects and Methods

The present study was conducted according to the ethical principles described in the Declaration of Helsinki (version, 2002 http://www.wma.net/en/30publications/10policies/b3/). The protocol of the study was approved by the Research Ethics Committee of the Dental School of the University of Athens, Greece (Protocol number 135/26.01.2010).

### 2.1. Participants

All patients with complete cleft lip and/or palate registered and treated in the Department of Orthodontics, University of Athens, Greece, from January 1994 to May 2010 were evaluated for inclusion in the present study. Subjects younger than 9 years or subjects with syndromes and other congenital anomalies with associated malformations, as well as subjects with moderate to severe mental retardation, were excluded from the study. The surgical treatment of the patients followed various protocols, but all patients received or were receiving orthodontic treatment at the time of evaluation at the Postgraduate Orthodontic Clinic of the University of Athens.

The potential study group consisted of 74 patients and their parents (father or mother). Slightly more than half of them were living in or near Athens, while the rest were spread in the whole Greek territory. Several attempts for phone contact were made within one month and communication was feasible with 51 patient families. From them, the response rate was 68.6%, which refers to 35 patients and 32 parents, all being of Caucasian origin. Two patient/parent pairs were excluded because of moderate mental retardation. Thus, the final sample consisted of 33 patients and 30 parents (Table 1). In the present study, nonparticipation is mainly attributed to difficulties in contacting patients (60.5% out of all nonparticipants). From the contacted patients that did not participate in the study, the reason is unknown for 3 out of 16.

On the day of examination, the subjects were thoroughly informed of the study and they signed an informed consent form. No patient/parent that agreed to participate on the first phone contact refused after that point. Each subject was included in the study with a code number in order to blind the method.

### 2.2. Interview-Guided Questionnaires

Two of the authors guided the individuals with cleft and their parents to fill in the questionnaire, in standardized sessions. Special consideration was taken so that the patients and their parents did not have any contact until both responded to the questionnaire, were not in the interview room at the same time, and were not familiar with their interviewer. The same investigator interviewed each patient-parent pair. In case the patient was accompanied by more than one parent, the selection of the parent to participate was made randomly by the interviewer.

The questionnaire (Table 2) was constructed especially for this case and proved to be both reliable (internal consistency) and valid (concurrent, convergent, discriminant, and statistical conclusion validity; data not shown in detail; see also statistical analysis section) for the Greek population used. Content validity was attained by the study of various questionnaires previously used in relevant research areas [3–11]. The questionnaire was constructed after thorough discussion among all authors [12].

The answers were registered on a 100 mm Visual Analogue Scale (VAS). The distances between the start of the scale (“0”) and the markings of each rater were measured (mm) with an electronic digital pointed jaw caliper (Jainmed Inc., Seoul, Korea) by one investigator to transform ratings to continuous metric variables (min: 0, max: 100).

### 2.3. Statistical Analysis

Data analysis was performed using SPSS (Statistical Package for the Social Sciences, version 17.0, SPSS Inc., Chicago, IL, USA). Because the three groups (CP, UCLP, and BCLP) consisted of dissimilar sample sizes and the Shapiro-Will test showed abnormal distributions of part of the data, nonparametric statistics were used. On the other hand, Levene's test revealed homogeneity of variances in all cases examined.

After evaluating intergroup differences in an exploratory manner and because sample size and composition considerations did not allow for a valid evaluation of each group separately, UCLP + BCLP individuals were treated as a single main study group. However, most analyses were performed twice, once for UCLP + BCLP group and a second time including also the CP group. This approach was followed because CP is considered a quite different condition from CLP and this could have a confounding effect on the results.

Internal consistency for patients' and parents' groups for each subscale (esthetic, function, and everyday life related questions) was measured through Cronbach's alpha (*α*) [13] for the UCLP + BCLP group. The effect of deleting each item at once from a subscale in the obtained alpha values was also examined. A level above 0.8 was considered high consistency, while above 0.7 was considered acceptable.

Intergroup reliability between parents and patients was calculated by means of Spearman correlation coefficient. This, along with comparative statistical tests, was used to test agreement between parents and patients and also as a test of reliability and an example of concurrent and statistical conclusion validity of the questionnaire.

The relation of patient's age with patients/parents satisfaction was also tested by means of Spearman correlation coefficient. Similar correlations were also performed to explore potential associations of esthetic and functional with everyday life parameters. The level above 0.7 is considered a high correlation in this study, while moderate correlation is defined between 0.4 and 0.7.

The alpha level was set at 0.05 for all tests.

### 2.4. Error of the Method

In order to calculate the error of measurements, 30 VAS scores were measured again by the same researcher two weeks after the initial measurement. Paired *t*-tests between the first and the second measurements and Dahlberg's method were used for the systematic and random error, respectively. No systematic error was found in the measurement of VAS scores. The random error ranged from 0.03 mm to 0.52 mm (mean value = 0.19 mm), which was considered acceptable.

## 3. Results

A detailed description of the 33 patients (7 CP, 20 UCLP, and 6 BCLP) and 30 parents analyzed in the study is provided in Table 1. All three cleft groups included individuals of similar age, ranging from early adolescence to young adulthood (median: 17.1; range: 9.0–33.1 yrs). The status of treatment of the included subjects was similar between CP and UCLP groups but different in BCLP group. However, when UCLP and BCLP groups were pooled, as in most analyses performed in the present study, treatment status distribution was similar to that of the CP group (Table 1), thus reducing the confounding effect of this parameter.

The internal consistencies of each of the three subscales (esthetic, function, and everyday life) were generally acceptable with a median Cronbach's alpha of 0.78. Only Cronbach's alpha regarding the assessments of everyday life subscale was found just below the 0.70 level of acceptance (0.65), but this concerned only parents group. Examination of the importance of each individual item of the specific subscale to the alphas revealed that eliminating any item from the everyday life subscale would not increase alpha values significantly and for both groups and so we decided to keep all items (Table 3).

**Figure 1:**
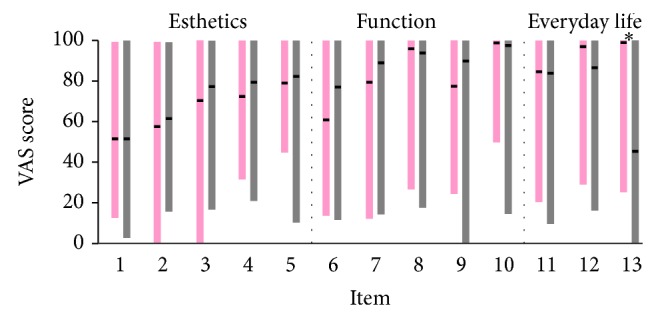
Bar graphs showing the responses of patients (pink color) and parents (grey color) for each item of the questionnaire. The upper limit of each bar represents the maximum value, the lower limit the minimum value, and the horizontal black line the median value. Asterisks indicate significant difference at *P* < 0.05 (Wilcoxon signed rank test). The vertical grey dashed line delimits the different subscales. No differences were detected in the results whether CP group was included or not in the analysis and therefore, for reasons described in the text, results for BCLP + UCLP group are shown for esthetic and everyday life parameters, while for function all three groups are included.

In general, there were no differences between the median parents' and patients' assessments in 12 out of 13 items tested. The only difference considered the influence of the cleft in family life. In contrast to their children, parents reported a significant impact of the cleft in family life, while they both assessed the impact of the cleft in social and professional/school life as minor. Both patients and their parents were moderately satisfied with the esthetics of the lips and the nose, while the situation improved when teeth, jaws, and face were considered. In general, patients and their parents were quite satisfied with function, with the exception of speech, mainly for patients. Results were similar whether the CP group was included or not but are only presented once including only the two CLP groups for esthetic and everyday life parameters and all three cleft groups for functional parameters, for reasons described previously (Figure 1).

On the other hand, interrater agreement between group pairs of patients and their parents was moderate to low (0.37 < rho < 0.43, *P* < 0.05) and detectable only for specific cases related to esthetics (Items 1, 3, 4) and function assessments (Item 8). Again results were similar whether the CP group was added to the UCLP + BCLP group or not and thus are only presented once (Table 4).

**Figure 2:**
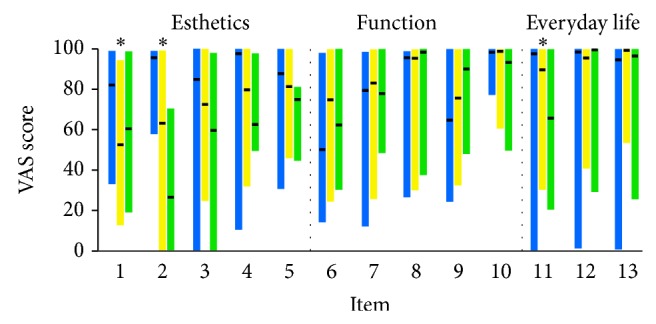
Bar graphs showing the responses of patients according to cleft type (CP: blue color, UCLP: yellow color, and BCLP: green color). The upper limit of each bar represents the maximum value, the lower limit the minimum value, and the horizontal black line the median value. Asterisks indicate significant difference at *P* < 0.05 (Kruskal-Wallis test followed by Mann-Whitney *U* test for pairwise comparisons). The vertical grey dashed line delimits the different subscales. Regarding item 1 (nose), significant difference was detected between CP and UCLP groups, while regarding item 2 (upper lip) all three groups differed significantly from each other. In item 11 (social activity), BCLP group was significantly different from the other two groups. Results for parents are similar and thus are not shown.

Although potential differences in the assessments of the three cleft groups were tested in an exploratory manner due to the small sample size of CP and BCLP groups, significant differences were evident for the esthetics of the lips and the nose and also for the effect of the cleft in social activity of the patients. The CP group was significantly more satisfied with the esthetics of the nose, as expected, followed by the BCLP and the UCLP group. Concerning lip esthetics, CP group was again the most satisfied but was followed by UCLP and finally BCLP group. The effect of the cleft on patients' social activity was significantly greater for the BCLP group compared to the other two groups (Figure 2).

Correlation analyses were also performed in an exploratory manner due to sample size and composition considerations. Concerning potential associations of patients' age with assessments, few significant and marginally nonsignificant correlations were detected and these were of moderate to low strength (0.31 < rho < 0.41, *P* < 0.10). However, it was interesting to note a tendency for improved satisfaction from speech and hearing with increasing age, while the opposite was true for lip esthetics. Furthermore, the influence of cleft on professional/school life was decreasing with age while the effect on social activity had an opposite tendency (Table 5).

Correlation analyses between esthetic and functional with everyday life parameters revealed various associations of moderate strength (0.35 < rho < 0.64, *P* < 0.05) between increased satisfaction from esthetic or functional parameters and decreased influence of the cleft in everyday life parameters. These mainly considered lip and nose esthetics and speech function, while dissatisfaction by all tested functions was correlated with increased influence of the cleft in professional/school life according to patients. Although significant differences were not evident in whether CP group was included or not, for clarity reasons, only UCLP + BCLP results are presented, while results based on all three groups are shown for function assessments (Table 6).

## 4. Discussion

Each step of the long-lasting and demanding treatment of an individual with cleft may play a vital role in the final esthetic and functional outcome and subsequently it may affect the everyday life of a person. Therefore, the evaluation of his/her satisfaction from aspects of treatment and the way these may affect everyday life is of great importance. The evaluation of treatment outcome in individuals with cleft is a difficult task because judgments are based upon certain criteria, which are not necessarily similar among different groups [14, 15]. Thus, the purpose of the present study was to assess the level of satisfaction of individuals with cleft and their parents by the esthetic and functional treatment outcome and test whether the results are associated with everyday life parameters and patient or treatment related characteristics. Indeed, significant associations were detected in several aspects and regarded mainly the esthetic result of treatment in the lips and the nose highlighting its crucial role for patients and their families. Speech impairment was another important parameter, where patients and parents reported reduced satisfaction, and was associated with increased influence in everyday life.

Overall, the responds of patients and parents were similar, with one exception concerning family life. On the contrary, interrater agreement was moderate to low and not detectable for all parameters. These findings indicate that although overall judgments are similar, when testing each patient/parent pair as a single case, previous experiences related to the cleft anomaly or other factors influence the two parts differently and can lead to bias [14]. Previous relevant studies by other researchers used qualitative scales for testing differences between patients' and parents' responses and reported contradictory findings [16–20]. To our knowledge, there is no similar study with the present methodology of quantitative assessments with VAS, although it has several advantages over qualitative assessments with categorical rating scales that have been presented elsewhere in detail [14, 15].

Among others, a primary advantage of the present study for the particular scientific field [10] is the comparison of three cleft types, even though the sample size in the CP and BCLP groups was relatively small. Of course, a better understanding of the differences among the three types of clefts and mainly the consequences that derive from these differences can be achieved by testing larger and more specifically defined samples. After evaluating intergroup differences in an exploratory manner, extensive differences were not evident between the UCLP and BCLP groups. Therefore, these two groups were pooled and treated as a single main study group. This resulted in an augmentation of the sample size for more valid evaluation of part of the tested hypotheses. The qualitative characteristics of these two groups were similar in regard to the presence of upper lip and nose defects, in contrast to the CP group which is not affected in these areas.

Another advantage of this study is the interview-based questionnaire with a high response rate of almost 70%. Also, nonparticipation was mainly attributed to the increased distance of the examination center from patient's or parent's residence. It is reported that the long treatment duration of cleft lip and/or palate is a deterrent factor for participation in research studies [21]. There is also general agreement that less satisfied or disappointed patients more often do not wish to participate in follow-up studies [21]. However, in the present study, the number of such cases, if so, is quite small (*n* ≤ 3). Previous interview-based studies reported a response of 46% to 58%, while the reasons for refusal were unknown for a significant part of nonparticipants [4, 22].

A potential limitation of the study could be the involvement of patients who completed any type of treatment and of those who were still in treatment. In accordance with previous studies, direct comparisons between these groups revealed a tendency for patients and their parents of the first category to report more positive assessments in esthetic, functional, and everyday life parameters [11, 18]. This could be attributed to the greater esthetic demands of people of older age or to the higher expectations of younger patients for future improvement through treatment. Further analyses of these results are not presented since sample size and composition considerations, along with age interference, do not allow for adequate control of confounding factors. The effect of this factor on the analyses was also indirectly tested with the investigation of correlations between age and patient/parent judgments. Indeed, patient age was found to affect the responses of the patients/parents in a few specific issues. For example, in the CP group, the level of satisfaction from the upper lip increased with age, although this particular group does not have cleft lip. This finding could be a random finding attributed to the relatively small sample size or a true finding for unaffected individuals. In contrast, the BCLP group, having a severely affected upper lip, presents a low level of satisfaction with upper lip esthetics which decreases further with age.

Evaluations of sex influence on assessments were not reported as significant between-group differences in cleft type, age, and treatment status distribution and the dissimilar association with the cleft condition could provide misleading results. In the UCLP group, males predominated, whereas in the BCLP and the CP groups, sex distribution was balanced. However, unpublished exploratory data from our study are in agreement with previous research that did not detect any significant difference between male and female participants [23–26].

Comparisons among the three cleft types resulted in significant differences mainly in the esthetics of the nose and the upper lip. Generally, dissatisfaction for the nose and upper lip esthetics in UCLP and BCLP groups is in concordance with previous studies [3–5, 18]. The CP group was significantly more satisfied with the esthetics of the nose, followed by the BCLP and the UCLP groups. Concerning lip esthetics, CP group was again the most satisfied but was followed by UCLP group and finally BCLP group. This seems reasonable when taking into consideration that UCLP patients have less scar tissue on the upper lip but more asymmetric nose compared to BCLP patients. The presence of scar tissue on the upper lips significantly affects facial esthetics [27], while several studies on noncleft groups reported an advantage for symmetric faces when judging facial attractiveness [28]. Indeed, studies on individuals with UCLP emphasized the importance of nose symmetry on facial esthetics [29, 30].

The effect of the cleft on patients' social activity was significantly greater for the BCLP group compared to the other groups and tended to increase with age. On the contrary, the influence of cleft on professional/school life was decreasing with age. Furthermore, there was a tendency for decreasing satisfaction from lip esthetics with age in this group, while the opposite was true for speech and hearing. These results seem quite reasonable since the reported improvement of speech/hearing is probably related to the decreasing influence of cleft on professional/school life. In a similar manner, but on an opposite direction, the reported decreasing satisfaction from lip esthetics can be related to the increasing influence of the cleft in patients' social activity. In agreement with our findings, a recent questionnaire study highlighted the negative influence of the cleft on patient's social activity and professional life in adults when poor esthetic results are evident [14], while another recent study in 5- to 9-year-old children with orofacial clefts found minor differences in psychosocial functioning compared with unaffected controls [31].

Apart from the esthetics of the lips and the nose, speech was the third major factor where patients and their parents reported reduced satisfaction and it was also correlated with increased influence of the cleft in everyday life. The level of satisfaction regarding speech in CP patients was found to increase significantly with age. It is probable that either they become familiar with the way they speak or they followed a successful speech therapy. In general, however, CP patients were less satisfied with their speech than UCLP and BCLP patients. This may happen because the latter cleft types have significant esthetic problems related to their cleft and as a consequence, they pay less attention to their speech. Finally, a statistically significant correlation was found between hearing and age in CP patients, even though this group is overall very satisfied. This finding may be associated with speech improvement with age or with the often ear infections of the younger CP patients.

It would be quite interesting for future prospective studies to examine more thoroughly patients' and parents' expectations from treatment at early ages and test the extent to which these are met at the completion of treatment and how this influences the psychosocial development and the everyday life of individuals with cleft.

## 5. Conclusions

Patients and parents compartmentalize the way they judge treatment, since they focus on the main component of their problem (according to cleft type) that does not seem to be adequately addressed by care providers. The present study highlights the need for satisfying lip and nose esthetic appearance for an undisturbed everyday life of individuals with cleft, especially regarding social interactions. Functional problems that are mainly related to speech impairment also affect social and professional/school life, but they seem to improve with age in certain cases. Future treatment strategies should focus on adequately addressing these issues with main treatment objective: the undisturbed everyday family life and social as well as professional functioning of individuals with cleft.

## Figures and Tables

**Table 1:** Overview of the patient sample characteristics regarding cleft type, age, sex, and treatment status distribution. Information for the available patient/parent pairs is shown in parentheses, since 3 patients do not have paired data with their parents.

Type of cleft	*n*	Sex	Age		Treatment status^**^	
			Median	Range	Complete	Incomplete
CP	7 (5)	4 M, 3 F (3 M, 2 F)	15.1 (14.8)	9.0–33.1	4 (2)	3
UCLP	20 (19)	15 M, 5 F (15 M, 4 F)	16.7 (16.4)	9.3–30.2	12 (11)	8
BCLP	6	3 M, 3 F	18.2	13.0–22.5	1	5

Total	33 (30)	22 M, 11 F (21 M, 9 F)	17.1^*^	9.0–33.1	17 (14)^***^	16

^*^Age did not differ significantly between the three groups (Kruskal-Wallis test).

^**^Treatment status refers to the entire treatment including revision surgery or speech therapy.

^***^The status of treatment of the subjects was similar in CP and UCLP groups but not in BCLP group. The status of treatment of the UCLP + BCLP group was similar to that of the CP group (chi-square test).

**Table 2:** Questions addressed to patients and their parents for the assessment of esthetics, function, and everyday life and 100 mm Visual Analogue Scale is used for each group of questions. “Not satisfied” and “Totally” correspond to 0. “Totally satisfied” and “Not at all” correspond to 100. When addressed to parents, Items 11 and 12 of Subscale C were adjusted in order to refer to their children's social activity and professional/school life, respectively.

Subscale A: Esthetics	

Item 1	What is your assessment regarding the esthetics of the nose?
Item 2	What is your assessment regarding the esthetics of the upper lip?
Item 3	What is your assessment regarding the esthetics of the teeth?
Item 4	What is your assessment regarding the esthetics of the jaws?
Item 5	What is your assessment regarding the esthetics of the face?

Subscale B: Function	

Item 6	What is your assessment regarding speech?
Item 7	What is your assessment regarding the level of being understood by other people during talk?
Item 8	What is your assessment regarding hearing?
Item 9	What is your assessment regarding respiration?
Item 10	What is your assessment regarding drinking ability?
	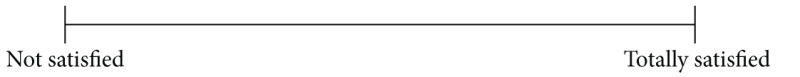

Subscale C: Everyday life	

Item 11	To what extent has the cleft influenced your social activity?
Item 12	To what extent has the cleft influenced your professional life or school activities?
Item 13	To what extent has the cleft influenced your family life?
	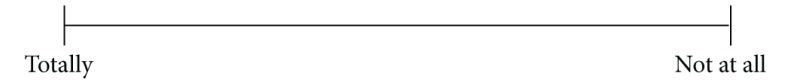

**Table 3:** Internal consistency of the answers of the UCLP + BCLP group for the three subscales of the questionnaire, measured by Cronbach's *α*, and influence of the deletion of each item by each subscale on Cronbach's alpha values.

Subscale	Items	Cronbach's alpha		
		Patients (if item deleted)	Parents (if item deleted)	Patients and parents mean (if item deleted)
Esthetics	All	0.827	0.773	0.818
	1	0.858^*^	0.742	0.809
	2	0.787	0.705	0.777
	3	0.769	0.757	0.799
	4	0.765	0.728	0.744
	5	0.779	0.724	0.780

Function	All	0.816	0.805	0.749
	6	0.733	0.701	0.614
	7	0.708	0.694	0.640
	8	0.814	0.880^*^	0.796^*^
	9	0.808	0.710	0.717
	10	0.808	0.797	0.724

Everyday life	All	0.782	0.650	0.747
	11	0.721	0.545	0.727
	12	0.598	0.408	0.523
	13	0.772	0.745^*^	0.728

^*^Cases where item deletion resulted in increased Cronbach's alpha value of the corresponding subscale.

**Table 4:** Interrater agreement between parents and patients for CP + UCLP + BCLP group measured by Spearman's correlation coefficient. Significant correlations are indicated by bold font. No significant differences were detected when this was tested on UCLP + BCLP group (not shown).

Subscale	Item	rho	*P*
Esthetics	1	**0.41**	**0.02**
	2	0.25	0.18
	3	**0.37**	**0.04**
	4	**0.43**	**0.02**
	5	0.06	0.74

Function	6	0.26	0.16
	7	0.27	0.14
	8	**0.39**	**0.03**
	9	0.19	0.32
	10	0.08	0.67

Everyday life	11	0.26	0.16
	12	0.16	0.40
	13	−0.07	0.72

**Table 5:** Significant or marginally nonsignificant correlations (Spearman's) of patients' or parents' answers with patients' age.

Item	Group	CP + UCLP + BCLP		UCLP + BCLP	
		rho	*P*	rho	*P*
2	Patients	−0.340	0.090	—	—
2	Parents	−0.412	0.024	−0.360	0.077
6	Patients	0.315	0.074	—	—
8	Parents	0.331	0.074	—	—
11	Parents	—	—	−0.351	0.085
12	Patients	0.382	0.028	0.389	0.049

**Table 6:** Spearman's correlations between satisfaction of patients and parents from esthetics (Items 1–5) and influence of the cleft on everyday life parameters (Items 11–13) for BCLP + UCLP group, and between satisfaction of patients and parents from function (Items 5–10) and influence of the cleft on everyday life parameters (Items 11–13) for CP + BCLP + UCLP group.

Item	Patients, rho (*P*)			Parents, rho (*P*)		
	11	12	13	11	12	13
1	—	—	—	—	0.400 (0.048)	0.573 (0.003)
2	0.444 (0.023)	—	—	—	—	—
3	—	—	—	—	—	—
4	—	—	—	—	—	—
5	—	—	—	—	—	—

6	0.350 (0.046)	0.453 (0.008)	—	0.523 (0.003)	0.430 (0.018)	0.467 (0.009)
7	0.420 (0.015)	0.433 (0.012)	—	0.457 (0.011)	0.379 (0.039)	0.396 (0.030)
8	—	0.415 (0.016)	—	—	—	—
9	—	0.640 (0.000)	—	—	—	0.541 (0.002)
10	—	0.428 (0.013)	—	0.537 (0.002)	0.435 (0.016)	0.429 (0.018)
